# Theoretical studies of hydrogen bond weakening of DNA base pairs using selective photon frequency radiation

**DOI:** 10.1039/d6ra02301k

**Published:** 2026-07-14

**Authors:** Yawen Li, Yan Jiang, Zhengfei Wen, Yuqi Xia, Yifan Guan, Zhe Wang, Kai Li, Peng Zhang

**Affiliations:** a Shandong Provincial Key Laboratory of Nuclear Science, Nuclear Energy Technology and Comprehensive Utilization, Weihai Frontier Innovation Institute of Nuclear Technology, School of Nuclear Science, Energy and Power Engineering, Shandong University Shandong 250061 China; b School of Space Science and Technology, Shandong University Weihai 264209 China; c Weihai Research Institute of Industrial Technology of Shandong University Weihai 264209 China

## Abstract

DNA, the quintessential carrier and transmitter of genetic information within living organisms, has consistently captivated the scientific community with its intricate structure and multifaceted functionality, making it a cornerstone of life sciences research. This paper introduces a theoretical analysis of vibrational spectra of DNA base pairs and explores a new photon radiation approach for hydrogen bond weakening. Through meticulous analysis of vibrational modes within the infrared spectrum, we have determined the optical frequencies that correspond to the vibrational frequencies between bases, aiming to induce a photon–phonon resonant absorption that could deposit energy into the hydrogen bond. Based on first-principles density functional theory (DFT) calculations, we have simulated the infrared spectra of the adenine–thymine (A–T) and guanine–cytosine (G–C) base pairs, successfully identifying the characteristic peaks in base pairs linked to hydrogen bond vibrations. We propose that infrared lasers at around 3200 cm^−1^ could resonantly excite these N–H modes, potentially depositing energy into the hydrogen bonds and thereby facilitating their weakening under appropriate conditions. Beyond advancing the understanding of vibrational dynamics in DNA base pairs, this work outlines a photon–phonon coupling concept that may inspire future experimental studies in nucleic acid manipulation. The theoretical framework presented herein provides a reference for further theoretical and experimental investigations of vibrational resonances in isolated base pairs, while acknowledging that practical implementation requires overcoming challenges related to the chemical environment.

## Abstract 2

DNA, the quintessential carrier and transmitter of genetic information within living organisms, has captivated the scientific community with its intricate structure, making it a cornerstone of life sciences research.

## Graphical abstract

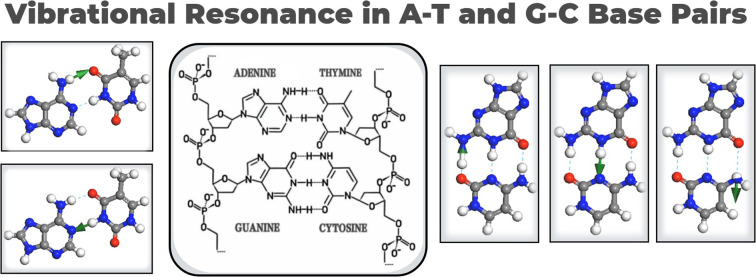

## 1. Introduction

**Fig. 1:**
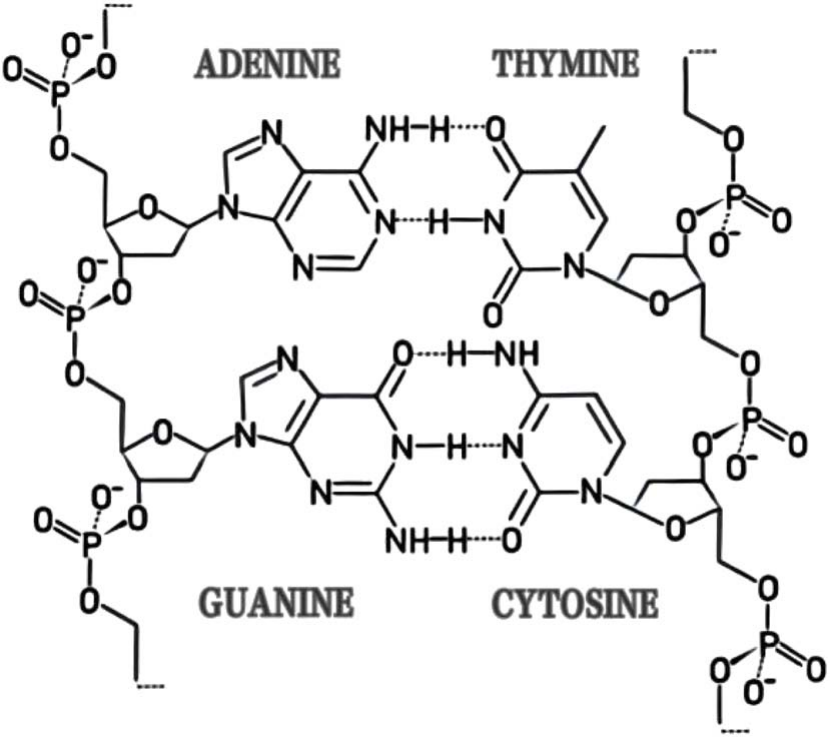
Structure diagram of a fragment of DNA molecule.

Deoxyribonucleic acid (DNA) is the cornerstone of life, responsible for carrying and transmitting genetic information within organisms. The studies of its structure, function, and roles in cellular biology, genetics, and molecular biology have always been a focal point in the field of life sciences.^1–4^ The seminal discovery of the double helix structure by Watson and Crick in 1953, known as the canonical B-form DNA, provided a foundational framework for biology.^1^ Meanwhile, subsequent research has revealed that DNA is a dynamic polymer capable of adopting a variety of alternative conformations, such as the A-form and Z-form, under different physiological conditions.^5^ Moreover, the stability of the double helix is governed by a complex interplay of forces, where Watson–Crick hydrogen bonding, π–π stacking interactions between adjacent bases, and the hydrophobic effect collectively ensure its structural integrity and function.^6^ The ongoing study of this structural diversity and its functional implications continues to be a central theme in molecular biology and biophysics.^7^ The applicability of the proposed the photon–phonon resonant absorption (PPRA) method, as developed and modeled in this work, is primarily tailored to the hydrogen bonding network of standard B-form DNA. In this work, the term “phonon” is used broadly to denote molecular vibrational modes in analogy to resonant coupling phenomena; traditionlly, “phonon” refers to collective lattice vibrations. Its efficacy when confronted with atypical structures, such as A-form or Z-form DNA, may be limited. The basic structural unit of a DNA molecule is the nucleotide, with each nucleotide consisting of a sugar molecule (deoxyribose), a phosphate group, and a base. The four types of bases are adenine (A), cytosine (C), guanine (G), and thymine (T). They are connected together according to certain pairing rules, where adenine pairs with thymine through double hydrogen bonds (A–T), and guanine pairs with cytosine through triple hydrogen bonds (G–C).^2,3^Fig. 1 illustrates the structure of a DNA molecule. This specific base-pairing rule is known as the Watson–Crick base pairing, which ensures the stability and replicability of the DNA molecule. The hydrogen bonds between the base pairs are one of the key factors maintaining the stability of the DNA double helix.

Scientists have attempted to unwind the DNA double strands to facilitate biological processes such as gene replication, transcription, and repair through various methods. These methods encompass enzyme-mediated unwinding,^4,8–11^ thermal unwinding,^12–16^ and chemical unwinding,^17–21^*etc.* enzyme-mediated unwinding utilizes DNA helicases to untangle the double strands. It is capable of recognizing DNA double strands and forming enzyme-DNA complexes, which can drive the unwinding process using the energy from ATP.^22,23^ Although enzyme-mediated unwinding methods are efficient and specific, they require enzyme involvement, and are operationally complex and costly.^24^ Thermal unwinding involves breaking the intramolecular hydrogen bonds by increasing temperature. The unwinding temperature is commonly referred to as the “melting temperature”, which depends on factors such as the sequence composition of DNA and environmental conditions.^25^ Thermal unwinding is operationally simple. However, precise temperature control is required, and the unwinding process cannot be directed, thus limiting its application.^26^ Chemical unwinding is achieved by breaking the structure of DNA molecules through the action of chemicals. These chemicals can be base pair-specific binders, such as “intercalators” and “minor groove binders”, which upon binding to specific regions of DNA molecules, cause changes in DNA structure, leading to the unwinding of DNA double strands.^27^ Chemical unwinding methods have been widely used in laboratory research. However, since they may damage DNA molecules, their potential in actual biomedical applications still needs further evaluation.^28^

To overcome the limitations of traditional DNA denaturation techniques, herein we propose a novel method based on photon–phonon resonant absorption (PPRA).^29^ This method is grounded in the well-established principle of infrared spectroscopy, wherein photons in the mid-infrared range (corresponding to wavenumbers from ∼500 to 4000 cm^−1^) can be resonantly absorbed by molecular systems when the photon energy matches the energy of a specific molecular vibration. This resonant energy transfer can, in principle, lead to bond rupture when the induced strain exceeds the bond's strength.^30^ The concept of using resonant infrared excitation to disrupt specific molecular bonds has been explored in other contexts,^31^ and its application to DNA is supported by experimental evidence that shows DNA exhibits characteristic absorption in the terahertz/far-infrared region, corresponding to collective vibrational modes of its structure.^32^ Recent experimental and theoretical studies have further supported this concept. Zhang *et al.* demonstrated that mid-infrared photons resonant with carbonyl stretching vibrations can significantly enhance DNA duplex unwinding during polymerase chain reaction, an effect that is non-thermal and can be amplified by using D_2_O as a solvent.^33^ In parallel, advanced modeling by Qian *et al.* revealed that demonstrate that transition dipole strengths of C=O and C=C modes are environment-independent, which supports our conclusions.^34^ This method simulated the vibrational properties between bases in DNA molecules to determine the laser frequencies that match the vibrational mode between bases to achieve the hydrogen bonds breaking. This theoretical framework identifies vibrational resonances that may, in principle, be relevant to energy deposition into hydrogen bonds. However, experimental validation and mechanistic demonstration remain beyond the scope of the present theoretical work.

## 2. Method

Due to the diversity and vast size of a DNA molecule, we only calculated the two base pairs independently to balance the computational cost. The initial hydrogen bond distances for the isolated base pairs were set based on high-level calculations on isolated base pairs in the gas phase using reference hydrogen bond distances of 2.88 Å and 2.80 Å for the A–T pair, and 2.89 Å, 2.88 Å, and 2.75 Å for the G–C pair for initial setup and comparison.^35^ These reference distances are taken from calculations on isolated gas-phase base pairs reported by Stasyuk *et al.*, representing the equilibrium hydrogen bond lengths for Watson–Crick pairs in the absence of environmental perturbations.^35^ The two different values for the A–T pair (2.88 Å and 2.80 Å) correspond to the two distinct hydrogen bonds: N–H⋯O (longer) and N–H⋯N (shorter), respectively. For the G–C pair, the three values (2.89 Å, 2.88 Å and 2.75 Å) correspond to the three hydrogen bonds with different donor–acceptor combinations (N–H⋯N, N–H⋯O and O⋯H–N). Utilizing first-principles density functional theory (DFT) simulations, we conducted geometric optimization and frequency calculations of both A–T and G–C base pairs with the DMol3 code.^36^ Šponer *et al.* demonstrated that the Becke3LYP DFT functional (a forerunner of B3LYP) overestimates the stabilization energy of stacked base pairs when used non-dispersion correction, and is not suitable for stacked systems.^37^ To account for dispersion interactions, we optimized all structures and calculated the vibrational frequencies using the B3LYP-D dispersion-corrected functional.^38^ And the basic set is dnp (double numerical plus polarization). The dnp basis set is the standard high-quality numerical basis set in the DMol3 code. We evaluated the performance of several exchange–correlation functionals for predicting the two hydrogen bond lengths in the A–T base pair. The B3LYP-D hybrid functional was selected for all subsequent calculations, as it provided the closest agreement with experimental data (see Table 1). This functional has been widely validated and is well-suited for modeling hydrogen-bonding interactions in biological systems.^39,40^ The convergence tolerance for the self-consistent field (SCF) was set to 1 × 10^−7^ eV per atom. The energy tolerance was set to 1 × 10^−7^ Ha. Based on the frequency calculations, the dynamic process of the normal modes at the gamma point was analyzed, and the peaks in the simulated infrared (IR) spectrum were assigned according to the corresponding vibrations. This allows us to analyze the peaks of the IR spectrum observed in reported experiments and identify the hydrogen bond related vibrations with high absorption intensities.

**Table 1:** Comparisons of different functionals with experimental data of two A–T hydrogen bonds. The unit of bond length is Å

	Hybrid-B3LYP-D	GGA-RPBE	GGA-BLYP	GGA-PBE	Ref. data
A–T bond 1	2.90	2.90	3.19	2.93	2.80
A–T bond 2	2.90	3.00	3.43	2.90	2.88

## 3. Results and discussion

**Fig. 2:**
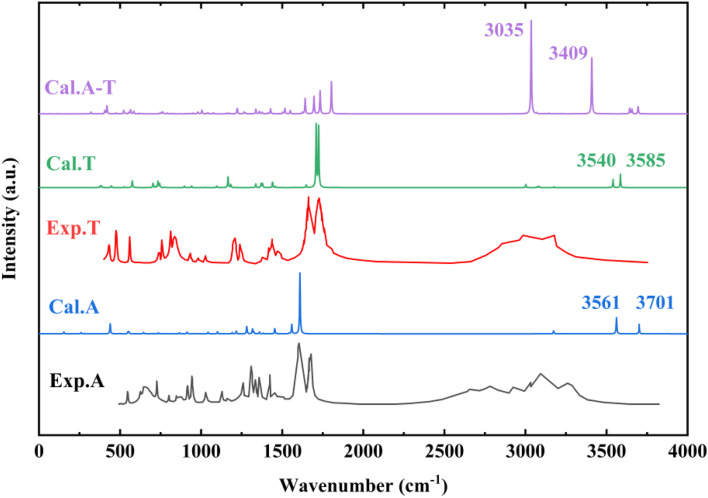
The calculated IR spectrum (purple) of the A–T base pair. And the calculated spectra of T (green) and A (blue) molecules together with their experimental data (red and black).

In the microscopic world, all the atoms in a crystal or in a molecule are in constant thermal vibrations at above absolute zero. For one atom, its totally vibrational mode is a combination of 3 × *n* −6 (*n* is the total number of atoms) normal modes, some of which can be detected by IR or Raman spectrometer subject to different selection rules. The simulated distribution intensity of all the detected normal modes along the frequency axis corresponds to the experimental IR or Raman spectrum, which is in the range of 0–4000 cm^−1^. The calculated IR spectrum of A–T base pairs is shown in Fig. 2. We also presented the calculated IR spectra of separate A and T molecules together with their experimental spectra for comparisons.^41^ Owing to the challenges in obtaining experimental IR spectra of base pairs in the gas phase and the corresponding lack of reference data, we used isolated nucleobases as a reliable and well-established benchmark to validate our computational method. This approach allows us to capture the key vibrational features to guide future experimental investigations of nucleobase vibrations.

**Fig. 3:**
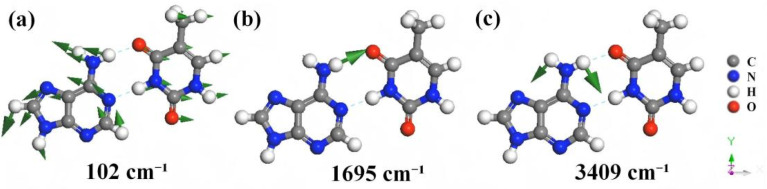
Three kinds of vibrational normal modes of the A–T base pair in different energy bands. (a) Cluster vibration mode; (b) NH_2_ bending mode; (c) NH_2_ stretching mode.

The dynamic process of each normal mode can be analyzed individually. Thus, the main peaks were assigned corresponding to a specific vibrational mode. We simply divided all the normal modes into three categories: cluster vibration, bending, and stretching. Three examples in different energy bands were shown in Fig. 3.

**Fig. 4:**
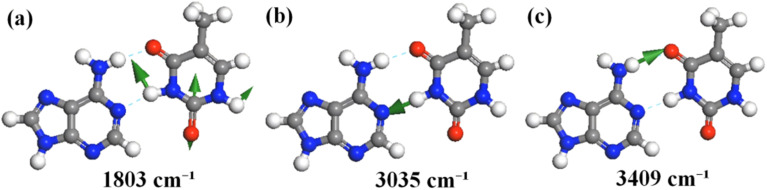
Three typical examples of vibrational modes of the A–T base. (a) Vibration mode of ring breathing at 1803 cm^−1^; (b) one hydrogen bond-related vibration at 3035 cm^−1^; (c) the other hydrogen bond-related vibration at 3409 cm^−1^.

Besides, the calculated frequency for the ring breathing mode is 1803 cm^−1^, as shown in Fig. 4(a), consistent with the experimental assignment at 1733 cm^−1^ that involves the conjugated C=O stretch coupled with N–H bending.^42^ Since the IR signal of hydrogen bond vibrations in the low energy region was quite weak, we focus on the related intramolecular N–H vibrations in the high energy region. The energy level transition of these vibrational modes would facilitate hydrogen bond breaking.

As shown in Fig. 2, it can be seen from the simulated IR spectrum that the two strongest absorption peaks are at 3035 cm^−1^ and 3409 cm^−1^. We presented simulated isolated A and T monomers together with their experimental spectra for comparisons. Both A and T have no matching strong peaks as the A–T base pair. For the monomer of T, Shang assigned a peak at 3201 cm^−1^ to the stretching vibration of the hydrogen-bonded N–H group in the macrocyclic cavity, which exhibits restricted mobility due to its coordination environment.^42^ In this work, the IR spectrum of isolated T exhibits two distinct N–H stretching bands at 3540 and 3585 cm^−1^. The N–H stretching mode at 3035 cm^−1^ of the A–T base pair is corresponding to the mode of T at 3540 cm^−1^. The large redshift of this mode shows the huge influence of hydrogen bonding between A and T.^43,44^ The N–H group of T acts as a hydrogen bond donor to the complementary acceptor site of A. This hydrogen bonding induces significant modifications in the vibrational characteristics of the N–H group. Specifically, the vibrational mode shifts from a pure stretching motion to a coupled mode involving concerted hydrogen bond stretching and bending motions. Such vibrational coupling leads to a substantial reduction in vibrational energy, manifesting as a pronounced redshift of the absorption peak to 3035 cm^−1^. The redshift of the N–H stretching frequency and its coupling to hydrogen bond vibrations observed in this work are characteristic features of strongly hydrogen-bonded systems. Please see the dynamic process of this mode from the SI file S2. This phenomenon aligns with the vibrational coupling and energy redistribution mechanisms revealed by advanced multidimensional spectroscopy in complex molecular systems.^30^ The vibrational mode depicted in Fig. 4(b) corresponds to the stretching vibration between nitrogen and hydrogen.

Colarusso *et al.* measured the gas phase A and observed a strong IR peak at 3434 cm^−1^, corresponding to the N–H stretching vibration.^45^ In this work, the isolated A exhibits two distinct N–H stretching vibrations at 3561 and 3701 cm^−1^. The lower-frequency mode is assigned to symmetric stretching vibration of the N–H group, whereas the higher-frequency one corresponds to asymmetric stretching, characterized by out-of-phase N–H bond displacements. The N–H of adenine acts as a hydrogen bond donor to the O atom of thymine. The hydrogen bonding induces a pronounced redshift of the N–H stretching frequency to 3409 cm^−1^ -a characteristic signature of strong hydrogen bonding in nucleic acid base pairs.^46^ Theoretical studies by Fisher, Walther and Jepsen have shown that such coupling involves concerted motions of the hydrogen bond donor and acceptor groups, leading to characteristic low-frequency absorption bands, as observed in the collective terahertz modes of biomolecules.^32^Fig. 4(c) revealed the N–H stretching vibration of A is along the hydrogen bond direction.

**Fig. 5:**
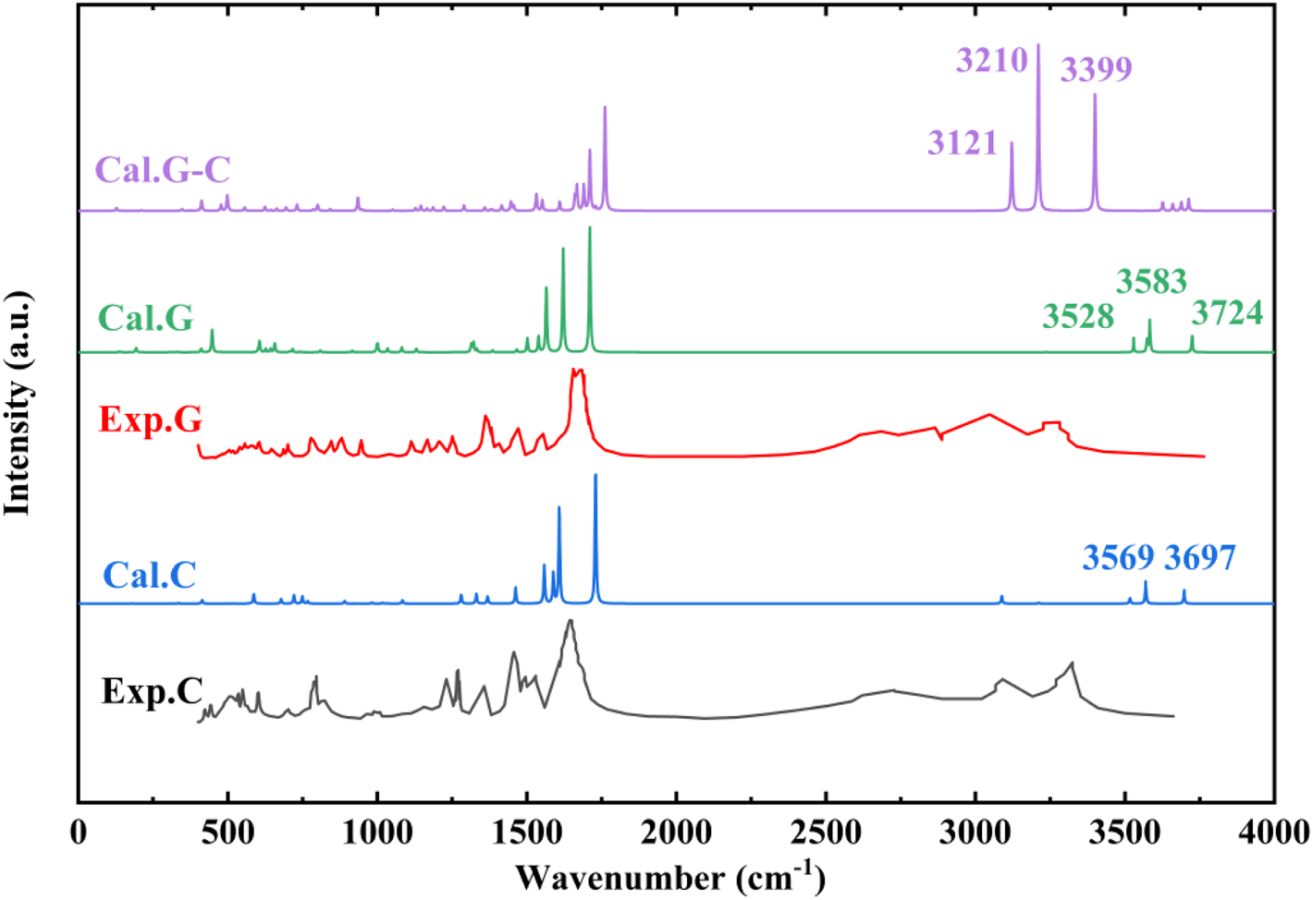
The calculated IR spectrum (purple) of the G–C base pair, followed by the calculated spectra of G (green) and C (blue) molecules together with their experimental data (red and black).

**Fig. 6:**
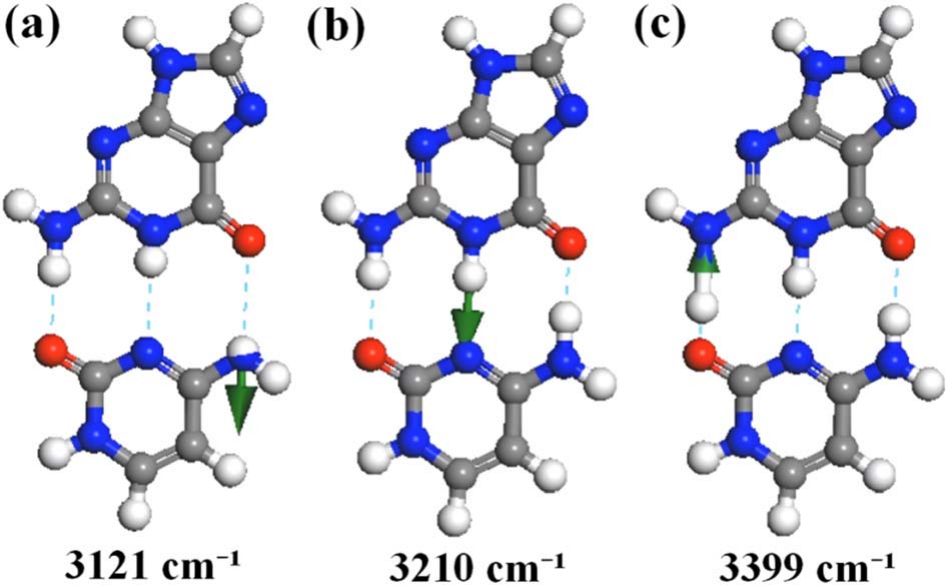
Three hydrogen bond-related vibrational modes of G–C base pairs. (a) One hydrogen bond-related vibration at 3121 cm^−1^; (b) at 3210 cm^−1^; (c) at 3399 cm^−1^.

Fig. 5 presents the calculated IR spectrum of G–C base pairs compared with the separate A and T molecules, as well as the experimental spectra.^41^ The G–C base pairs showed very strong peaks located at 3121, 3210 and 3399 cm^−1^, respectively. According to the normal mode analysis, these three vibrational modes correspond to the N–H stretching vibrations of cytosine, the guanine ring, and the amino group of guanine. As shown in Fig. 6, all of these vibrations are directly connected with hydrogen bonds.

For isolated C, the NH_2_ group exhibits two distinct stretching vibrations at 3569 and 3697 cm^−1^. The lower-frequency mode corresponds to the symmetric stretching vibration (in-phase N–H motions), while the higher-frequency peak is assigned to the asymmetric stretching mode (out-of-phase N–H motions). Upon forming the G–C base pair, the N–H group participates in a hydrogen bond with the O atom of G, resulting in a significant redshift from 3569 to 3121 cm^−1^. This shift arises from mechanisms of vibrational coupling and energy redistribution,^30^ as shown in Fig. 6(a).

The isolated G exhibits distinct N–H vibrational signatures: a N–H stretching mode at 3528 cm^−1^ (assigned to the free N–H group) and two NH_2_ stretching vibrations at 3583 cm^−1^ (symmetric, in-phase) and 3724 cm^−1^ (asymmetric, out-of-phase). Upon Watson–Crick pairing theory with C, these modes undergo significant perturbations: acts as a hydrogen bond donor to the N atom of C, resulting in a pronounced red shift from 3528 to 3210 cm^−1^. Please see the dynamic process of this mode from the SI file S2. The vibrational mode is illustrated in Fig. 6(b). One N–H group forms a hydrogen bond with the O atom of C, shifting the symmetric stretching frequency from 3583 to 3399 cm^−1^ as shown in Fig. 6(c). The observed redshifts originate from two synergistic effects. One is the electronic redistribution. The hydrogen bonding reduces the N–H bond force constant, lowering the vibrational energy. The other one is vibrational coupling. The stretching motions couple with collective modes along the hydrogen bond axis. These results underscore the universal role of hydrogen bonding in modulating nucleic acid vibrational dynamics.^30,46^

The simulated IR spectra of A–T and G–C base pairs revealed characteristic vibrational modes associated with hydrogen bonds. For the A–T pair, two prominent absorption peaks were identified at 3035 and 3409 cm^−1^, corresponding to the N–H stretching vibrations along the hydrogen bond direction. Similarly, the G–C pair exhibited three characteristic peaks at 3121, 3210, and 3399 cm^−1^, which are attributed to N–H stretching modes in the isolated C or G. The results highlight the importance of the exchange–correlation functional in modeling weak interactions such as hydrogen bonds. Notably, the redshift observed in the A–T pair (*e.g.*, from 3540 cm^−1^ to 3035 cm^−1^) reflects the weakening of hydrogen bonds, a critical precursor to bond rupture.

Take the vibrational mode approximately as a simple harmonic oscillator; the vibrational frequency is:
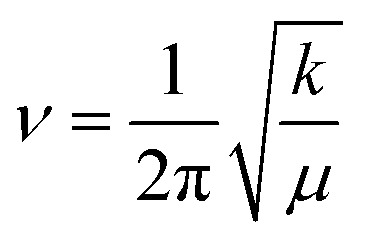
where *k* represents the force constant, and *µ* denotes the reduced mass. For a vibrational mode with frequency *ν*, the vibrational energy is:
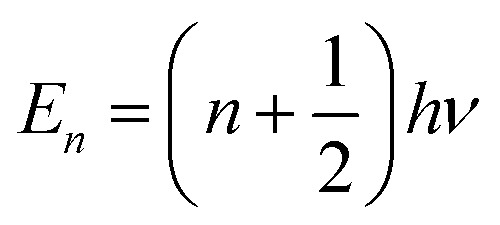


When the vibrational mode absorbs a photon with frequency *ν*, it excites a normal mode corresponding to this specific frequency, *i.e.*, the *n* is from 0 to 1. Taking the peak observed at 3035 cm^−1^ as an example, its zero-point vibrational energy is *E*_0_ = 188 meV. Assuming it absorbs a photon at this resonant frequency, the energy of the excited state increases to *E*_1_ = 564 meV. This energy is comparable to typical hydrogen bond interaction energies of isolated base pairs, which are approximately 506 meV for A–T.^47^ For low energy levels (*n* = 0, 1, 2), the energy interval is nearly harmonic, and the vibrational frequencies obtained from harmonic DFT calculations are reliable for describing the *n* = 0 → 1 transition. Therefore, our calculations identify a resonance condition (energy match between a photon and an N–H stretching mode) and suggest a hypothesis that such excitation might contribute to energy deposition. When we provide a photon at the corresponding frequency, since the vibrational kinetic energy of the hydrogen atom exceeds the hydrogen bond energy, absorption of one photon at this frequency may break the intermolecular hydrogen bond. To quantify the impact of the photon–phonon resonance, we analyzed the relative IR intensities obtained from DFT calculations. The peak at 3200 cm^−1^ (near the G–C vibrational frequencies) demonstrated the highest relative IR intensity, indicating favorable resonance conditions (the calculated mode intensity at 3210 cm^−1^ is 1040 km mol^−1^, while the adjacent modes at 3160 cm^−1^ and 3260 cm^−1^ have intensities of 6.48 and 3.07 km mol^−1^). In this work, we restrict our conclusions to the identification of resonant frequencies and the possibility of energy transfer. By applying IR lasers at this frequency, the energy transfer from photons to normal modes induces vibrational excitation, effectively reducing the activation energy for hydrogen bond rupture. The identified vibrational frequencies (3035, 3121, 3210, 3399, and 3409 cm^−1^) may provide a targeted framework for experimentalinvestigations of vibrational resonances in isolated base pairs.

The proposed frequencies (3035–3399 cm^−1^) overlap with the broad O–H stretching band of liquid water (center ∼3400 cm^−1^).^48^ In aqueous media, water dominates absorption and competes for IR energy. A rough estimate: the first hydration shell of DNA contains ∼20–30 water molecules per base pair,^49^ and the water O–H absorption band ranges from 3200 cm^−1^ to 3600 cm^−1^. Whether the energy level transition of base-pair N–H modes can be achieved in aqueous environments and whether the excited modes retain sufficient localization and lifetime to influence bond breaking before IVR occurs? It remains an open question that would require explicit experimental validation. Thus, we offer our results based on vibrational assignment as a hypothesis for future testing. A full quantitative assessment of required laser influence is beyond this work.

For instance, tunable IR lasers such as free electron lasers could be calibrated to these frequencies to probe the vibrational excitations identified here in isolated nucleobase or base-pair systems. We have proposed this kind of PPRA technology for fat removal by rapid liquefaction of adipocytes.^29^ However, due to the physical background, we cannot perform such a biological macromolecule experiment. Hopefully, this theoretical work can arouse the interest of biological scientists in conducting experimental tests. Our calculations are restricted to isolated A–T and G–C base pairs in the gas phase under static (0 K) conditions. A real DNA duplex involves additional stabilizing factors (multiple hydrogen bonds, base stacking, backbone constraints, counterions, and solvent effects) that can alter vibrational spectra and the free-energy landscape for base-pair opening.^50,51^ Thus, direct extrapolation to double-stranded DNA unwinding would require further modeling of these complexities. Moreover, our analysis does not account for time-dependent dynamics, vibrational lifetimes, intramolecular vibrational energy redistribution (IVR), or coupling to the environment. In biomolecular systems, resonantly excited local modes typically relax on picosecond or sub-picosecond timescales *via* IVR. For instance, IVR following NH stretching excitation in adenine-uracil base pairs occurs on a sub-picosecond timescale, with hydrogen bond lifetimes of ∼0.5 ps;^52^ and mode-selective redistribution from NH stretching to bending modes in hydrogen-bonded systems shows sub-picosecond population transfer followed by relaxation to lower-frequency modes within ∼3 ps.^53^ These timescales suggest that coherence may be lost before significant energy accumulates along the hydrogen bond. Hence, whether the observed resonance condition can drive bond rupture requires further experimental verifications.

To avoid overinterpretation, we explicitly distinguish three levels of inference in this work. First, our DFT calculations reliably identify the hydrogen-bond-related IR-active modes, supported by comparison with experimental spectra of isolated nucleobases and by the characteristic redshifts upon base-pair formation. Second, the comparison of the vibrational energy of the harmonic oscillator and the hydrogen bond energy suggests a hypothesis that resonant mid-IR excitation might deposit energy into the hydrogen-bond. Third, the present calculations do not establish any mechanism by which such vibrational excitation leads to hydrogen-bond rupture. Therefore, we offer them as a vibrational assignment that may inform future experimental or computational investigations.

## 4. Conclusions

In this study, we have performed a theoretical analysis of vibrational modes in isolated A–T and G–C base pairs by integrating first-principles DFT. Key modes of hydrogen bond-related vibrations in A–T and G–C base pairs were identified at 3035, 3121, 3210, 3399, and 3409 cm^−1^. These frequencies, particularly the dominant peak at 3200 cm^−1^, were shown to induce coherent vibrational excitation through IR laser irradiation, potentially facilitating bond weakening under appropriate conditions.

The work outlines a photon-vibrational coupling concept for nucleic acid base pairs that may inspire future experimental investigations to circumvent the limitations of enzyme dependency, chemical toxicity, and thermal randomness. However, whether such an approach could ultimately address the limitations of existing DNA processing methods remains an open question requiring further study. Future research may focus on experimental validation using free electron lasers and on extending the theoretical framework to more realistic DNA models.

## Author contributions

Y. W. L. is mainly responsible for model construction and article writing, Y. J. and Z. W. assisted structural modeling and data processing; Y. X. and Y. G. helped to analyze the data; Z. W. and K. L. participated in the results discussion; P. Z. conducted simulations, data analysis, and edited the manuscript. All authors have read and agreed to the published version of the manuscript.

## Conflicts of interest

There are no conflicts to declare.

## Note added after first publication

This article replaces the version published on 14 July 2026, which included an error in one of the equations.

## Supplementary Material

RA-OLF-D6RA02301K-s001

RA-OLF-D6RA02301K-s002

## Data Availability

The datasets used during the current study are available from the corresponding author on reasonable request. Supplementary information (SI) is available. See DOI: https://doi.org/10.1039/d6ra02301k.
