# Supplementary figures and images for: Theoretical studies of hydrogen bond weakening of DNA base pairs using selective photon frequency radiation
Source: RSC Adv. 2026 Jul 14. Online ahead of print. doi: 10.1039/d6ra02301k (PMC13366174; doi:10.1039/d6ra02301k)

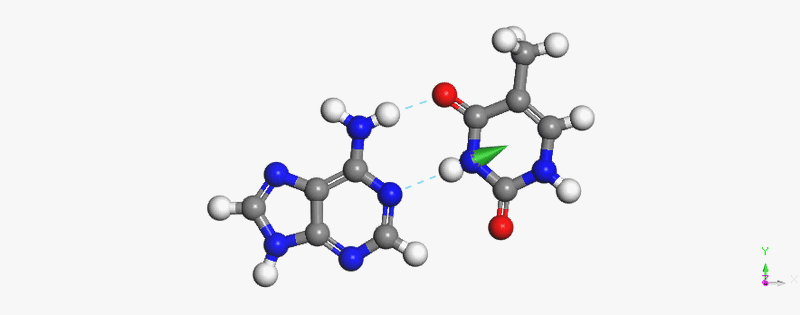

Supplement: RA-OLF-D6RA02301K-s001 [file RA-OLF-D6RA02301K-s001.gif]

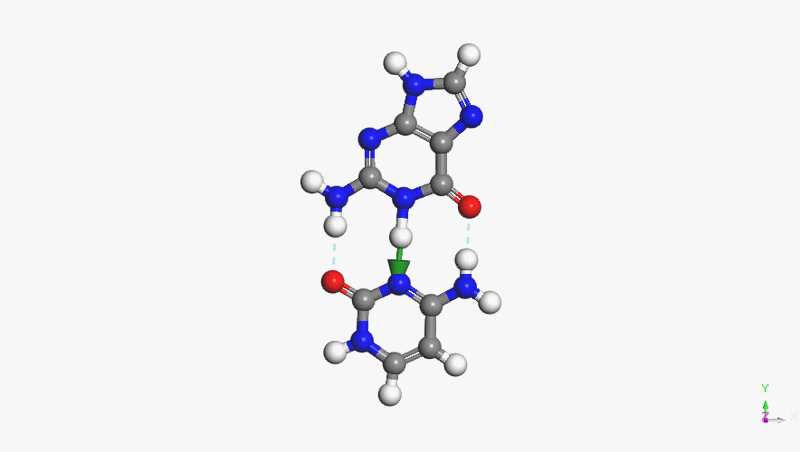

Supplement: RA-OLF-D6RA02301K-s002 [file RA-OLF-D6RA02301K-s002.gif]
